# Aerosol Characteristics and Physico-Chemical Compatibility of Combivent^®^ (Containing Salbutamol and Ipratropium Bromide) Mixed with Three Other Inhalants: Budesonide, Beclomethasone or N-Acetylcysteine

**DOI:** 10.3390/pharmaceutics12010078

**Published:** 2020-01-17

**Authors:** Rui Zhang, Junhua Hu, Liangjun Deng, Sha Li, Xi Chen, Fei Liu, Shanping Wang, Khaja Shameem Mohammed Abdul, Huimin Beng, Wen Tan

**Affiliations:** 1 Institute of Biomedical and Pharmaceutical Sciences, Guangdong University of Technology, Guangzhou 510006, Guangdong, China; scut_gdut@163.com (R.Z.); hu_jun_hua@163.com (J.H.); dengliangjun0127@163.com (L.D.); aya26139715@163.com (S.L.); ann_chen0806@163.com (X.C.); 1111706007@mail2.gdut.edu.cn (F.L.); khajashameem@gdut.edu.cn (K.S.M.A.); benghuimin@126.com (H.B.); 2 School of Bioscience and Bioengineering, South China University of Technology, Guangzhou 510006, Guangdong, China; shanpingwang@outlook.com

**Keywords:** inhalation, nebulizer, physico-chemical, particle size and distribution, delivery compatibility, Combivent^®^, Budesonide, Beclomethasone, *N*-acetylcysteine

## Abstract

Inhalation therapy with a nebulizer is widely used in chronic respiratory disease. Mixing inhalation solutions/suspensions for simultaneous inhalation is more convenient and might simplify the administration procedure. However, there are no data available to address the in vitro aerosol characteristics and physico-chemical compatibility of Combivent^®^ (containing Salbutamol and Ipratropium bromide) with other inhalation solutions/suspensions. In order to investigate the in vitro aerosol characteristics and physico-chemical compatibility of Combivent^®^ with Budesonide, Beclomethasone, and *N*-acetylcysteine, the appearance, pH, osmotic pressure, chemical stability, mass median aerodynamic diameter (MMAD), fine particles fraction (FPF), particle size corresponding to X50 (particle size, which accounts for 50% of the total cumulative percentage of volume of all particles), delivery rate, and total delivery of the mixed inhalation solution/suspension were tested. There was no change in the appearance such as a change in color or precipitation formation at room temperature. The pH, osmolality, and chemicals of the mixtures were stable for 24 h after mixing. There were no significant differences between Combivent^®^, Budesonide, Beclomethasone, *N*-acetylcysteine, and the mixtures in MMAD, FPF, X50, the delivery rate, and the total delivery. This indicates that the mixtures were physically and chemically compatible. The mixing did not influence the particle size, distribution, or delivery compatibility of the mixtures.

## 1. Introduction

Inhalation refers to a type of drug delivery system that transmits the drug of interest to either the respiratory tract or lungs in the form of an aerosol through a specific device to exert local or systemic effects. It has the advantage of fast onset delivery and no liver first-pass effect. In addition, due to the relatively low level of proteolytic enzymes in the lungs, inhalation is gradually used for the delivery of biological drugs such as proteins [1]. Inhalation-based drug delivery systems have been used for decades, mainly for the treatment of respiratory diseases such as asthma, chronic obstructive pulmonary disease (COPD), and pulmonary fibrosis [2,3]. There are three main types of inhalation: a metered-dose inhaler, dry powder inhaler, and nebulization [4]. Nebulization is the first inhalation form to deliver active drugs to the lungs in the form of aerosols [5]. Currently, it is one of the widely used inhalation forms in China [6]. Compared with the other two types of inhalation forms, it can be used in children, the elderly, those with disease, weakness, coma, and patients with poor coordination; it is simple to use and has good patient compliance [7,8].

Mixing different inhalation solutions/suspensions could simplify the administration procedure and time. However, simultaneous nebulization of an inhalation solution/suspension may affect drug delivery by altering the aerosol particle size distribution. It has been reported that Salbutamol and Ipratropium bromide can be mixed with cromolyn sodium, budesonide, and Tobramycin. Cromolyn is incompatible with Benzalkonium chloride [9,10]. The aerodynamic characteristics of procaterol may be affected by the mixture of procaterol and budesonide, but not budesonide [11]. The appearance, pH, and content of Levalbuterol mixed with Ipratropium bromide, Cromolyn Sodium, Acetylcysteine sodium, and Budesonide showed that the mixed inhalation solutions/suspensions were stable for at least 30 min [12]. Moreover, formoterol has physico-chemical compatibility with Ipratropium bromide, Acetylcysteine, and Budesonide aerosols [13]. The mixture of Fluticasone propionate, Ipratropium bromide, and Salbutamol sulfate had physico-chemical compatibility within 5 h. It was suggested that the mixture should be used immediately after mixing [14]. It is also suggested that Pulmozymel should not be mixed with Atrovent1LS and Sultanol [15].

Combivent^®^ (which contains Salbutamol and Ipratropium bromide) [16], Budesonide [17], and Beclomethasone [18] are widely used in the clinical treatment of asthma and COPD, and *N*-acetylcysteine is mainly used to remove airway mucus [19]. pH, osmotic pressure, and chemical stability are the main indexes for inhalation solution/suspension. Delivery compatibility and particle size distribution are the key quality attributes of inhalants [20]. In the clinic, Combivent^®^ is often used in combination with three other inhalation solutions/suspensions. However, there are no reports that address in vitro physico-chemical compatibility, particle size distribution, and drug delivery compatibility of these mixtures [9]. The current study will investigate these issues in detail, and the results may provide some insights for the use of medications in clinical practice.

## 2. Materials and Methods

### 2.1. Materials

The materials used for this study were potassium dihydrogen phosphate (Aladdin, Shanghai, China), acetonitrile (Thermo Fisher, Waltham, MA, USA), Combivent^®^ (an inhalation solution of 2.5 mL per dose; each dose contains 0.500 mg Ipratropium bromide and 3.000 mg Salbutamol sulfate; Boehringer Ingelheim, Ingelheim am Rhein, Germany), Pulmicort^®^ (an inhalation suspension of 2 mL per dose, with each dose containing 1 mg Budesonide; AstraZeneca, Cambridge, UK), Fluimucil^®^ (an inhalation solution of 3 mL per dose, with each dose containing 0.3 g *N*-acetylcysteine; Zambon, Bresso, Italy), and Clenil^®^ (an inhalation solution of 2 mL per dose, with each dose containing 0.8 mg Beclomethasone; Chiesi Farmaceutici, Parma, Italy). The instruments used for the current study were a nebulizer (BOY N, LC Sprint, PARI GmbH, Starnberg, Germany), respiratory simulator (BRS 2000, Copley Scientific Company, Nottingham, UK), next-generation impactor (NGI, Copley Scientific Company, Nottingham, UK), NGI pump (LCP5, Copley Scientific Company, Nottingham, UK), laser diffraction particle size analyzer (Sympatec, Clausthal-Zellerfeld, Germany), high-performance liquid chromatograph (HPLC, e2695-2489, waters), ssmometer (Model 5600, Norwood, MA, USA), and pH meter (LAQUAtwin, Kyoto, Japan).

### 2.2. Analysis of Physico-Chemical Compatability

Visual precipitation and color variation, the pH value, osmotic pressure, and content of three mixtures (Combivent^®^ and Pulmicort^®^; Combivent^®^ and Clenil^®^; Combivent^®^ and Fluimucil^®^) and the four single inhalation solutions/suspensions were tested, and samples were collected at 0 min, 30 min, and 24 h time intervals at room temperature. We used the inhalation solution/suspension clinical dose (Combivent^®^ 2.5 mL, Pulmicort^®^ 2 mL, Clenil^®^ 2 mL, Fluimucil^®^ 3 mL) as the test dose.

The HPLC method used for the Salbutamol, Ipratropium bromide and *N*-acetylcysteine content test was as follows: chromatograph: Waters e2695 column (Milford, MA, USA): Agilent TC-C18 (150 × 4.6 mm, 5 mm), wavelength: 210 nm, flow rate: 1.0 mL/min, injection volume: 20 mL, column temperature: 25 °C, mobile phase. Potassium dihydrogen phosphate solution was used as mobile phase A (2.50 g of potassium dihydrogen phosphate was dissolved in 800 mL water. The pH value was adjusted to 3.20 ± 0.05 with phosphoric acid solution and made to a constant volume of 1 L); acetonitrile was used as mobile phase B. The gradient conditions are shown in Table 1. The HPLC gradient conditions of the Salbutamol, Ipratropium bromide and Budesonide and Salbutamol, and Ipratropium bromide and Beclomethasone content tests are shown in Table 2. A double channel-detecting wavelength was adopted: 210 nm (Salbutamol and Ipratropium bromide) and 240 nm (Budesonide and Beclomethasone); the other HPLC methods were similar to those used for Combivent and *N*-acetylcysteine.

### 2.3. Measurement of Particle Size and Distribution

#### 2.3.1. Cascade Impactor Method

The NGI equipment was connected to the NGI pump and checked for air tightness. NGI was maintained at 5 °C for 2 h, and the flow rate was adjusted to 15 ± 5% L/Min before the test. Nebulization of the inhalation solution/suspension was measured with the test dose proportional to the clinical dose, and the accuracy of NGI detection was ensured. The test dose of each drug was as follows: Combivent^®^ 3 mL, Pulmicort^®^ 2.4 mL, Clenil^®^ 2.4 mL, Fluimucil^®^ 3.6 mL. Drugs were collected on each plate at specific time points during the test with methanol/water (1:1), and the contents were analysed with HLPC. All single inhalation solutions/suspensions and mixtures were tested three times. The mass median aerodynamic diameter (MMAD) and fine particle fraction (FPF) of the drug contents were also obtained from the software during the test.

#### 2.3.2. Laser Diffraction Method

Particle size parameters were analysed using the laser diffraction method. The equipment was initially preheated for 30 min, and test conditions were set as follows: lens, R2 (0.25/0.45–87.5 μm), trigger condition: refractive index ≥3%. After checking the background, test lasted for 120 s (test dose mixed according to the clinical dose: Combivent^®^ 2.5 mL, Pulmicort^®^ 2 mL, Clenil^®^ 2 mL, Fluimucil^®^ 3 mL). After completion of the test, we obtained the corresponding data X10 (particle size, which accounted for 10% of the total cumulative percentage of the volume of all particles), X50, X90 (particle size, which accounted for 90% of the total cumulative percentage of the volume of all particles) and span ($Span=\frac{X90-X10}{X50}$).

### 2.4. Delivery Rate and Total Delivery

To obtain the delivery rate and total delivery, a breathing simulator was connected with a filter membrane placed inside. The respiration simulation test is greatly influenced by the nebulization volume. When single inhalation solutions/suspensions were tested, we maintained a constant volume using saline. The mixed inhalation solutions/suspensions were 2 mL Combivent^®^ + 1.6 mL saline, 2 mL Combivent^®^ + 2.4 mL saline, 1.6 mL Pulmicort^®^ + 2 mL saline, 1.6 mL Clenil^®^ + 2 mL saline, 2.4 mL Fluimucil^®^ + 2 mL saline, 2 mL Combivent^®^ + 1.6 mL Pulmicort^®^, 2 mL Combivent^®^ + 1.6 mL Clenil^®^, 2 mL Combivent^®^ + 2.4 mL Fluimucil^®^. The breathing pattern used followed the USP adult breathing patterns recommended in the pharmacopoeia. The tidal volume was 500 mL, frequency was 15/min, and the duty cycle (t_i_/t_total_, the inhalation time divided by the period) was 1:1. A new filter membrane was used after 60 s collection, and nebulization continued until the nebulization was complete when the solution reached dryness. In order to prevent the saturation of the filter membrane, if necessary, the nebulization was interrupted to replace a new filter membrane (for the single inhalation solution/suspension mixed saline group and mixed group: one filter membrane was used in 1 min, and two new filter membranes were added until nebulization was complete).

The tested drug samples that were deposited on the filter membrane and filter device were extracted using sterile water. The drug was collected on filter membranes and the filter device with methanol water (1:1), and contents were tested by HPLC. The amount of drug collected by the first filter membrane divided by the collection time was defined as the delivery rate. The total amount of medicine collected by all filter membranes and the filter device was considered as the total delivery.

## 3. Results

### 3.1. The Physico-Chemical Compataibility

Appearance: There was no visual evidence of precipitation or physical incompatibility in any mixture at 0 min, 30 min, or 24 h. The appearance of Pulmicort^®^, Clenil^®^ and their mixtures with Combivent^®^ had slight cloudiness which is attributed to Pulmicort^®^ and Beclomethason in the suspension.

pH and Osmotic pressure: The change percentages of the pH value and osmotic pressure of the three mixtures of 30 min vs. 0 min and 24 h vs. 0 min were less than 3% (Table 3 and Table 4).

**Figure 1:**
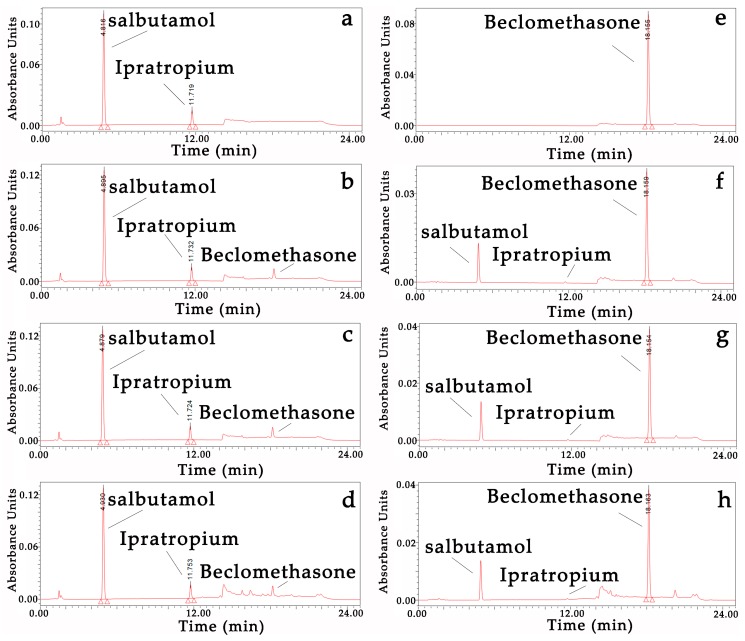
HPLC chromatogram of the three mixtures and single inhalation solutions/suspensions. (**a**) Salbutamol and Ipratropium bromide control (210 nm); (**b**) Salbutamol and Ipratropium bromide + Beclomethasone at 0 min (210 nm); (**c**) Salbutamol and Ipratropium bromide + Beclomethasone at 30 min (210 nm); (**d**) Salbutamol and Ipratropium bromide + Beclomethasone at 24 h (210 nm); (**e**) Beclomethasone control (240 nm); (**f**) Salbutamol and Ipratropium bromide + Beclomethasone at 0 min (240 nm); (**g**) Salbutamol and Ipratropium bromide + Beclomethasone at 30 min (240 nm); (**h**) Salbutamol and Ipratropium bromide + Beclomethasone at 24 h (240 nm).

Drug stability: There were no new chromatogram peaks in the mixtures (Figure 1), and the content change percentages of the three mixtures of 30 min vs. 0 min and 24 h vs. 0 min and the single inhalation solution/suspension vs. mixtures at three time points were less than 10% (Table 5).

### 3.2. Measurement of Particle Size and Distribution

#### 3.2.1. Method of the Cascade Impactor

**Figure 2:**
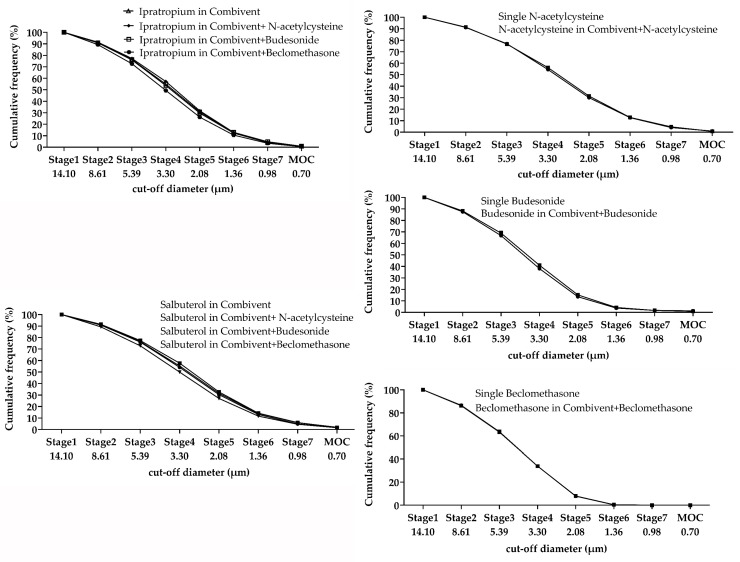
Cumulative frequency curve of single and mixed inhalation solutions.

The MMAD and FPF change percentages of the three mixtures compared to single inhalation solutions/suspensions were less than 10% (Table 6); the trend of the cumulative frequency curve was similar (Figure 2).

#### 3.2.2. Laser Diffraction Method

From Table 7 we can see that the X10 of single and mixed inhalation solutions/suspensions less than 1 µm, X50 around 3 µm, X90 around 9 µm, moreover, the span value was small, i.e., approximately 3. However, the X50 measured by laser diffraction method variation was relatively greater for Combivent® mixed with the two inhalation suspensions. And the X50 of the mixture with two suspensions tended to increase slightly.

### 3.3. Delivery Rate and Total Delivery

**Figure 3:**
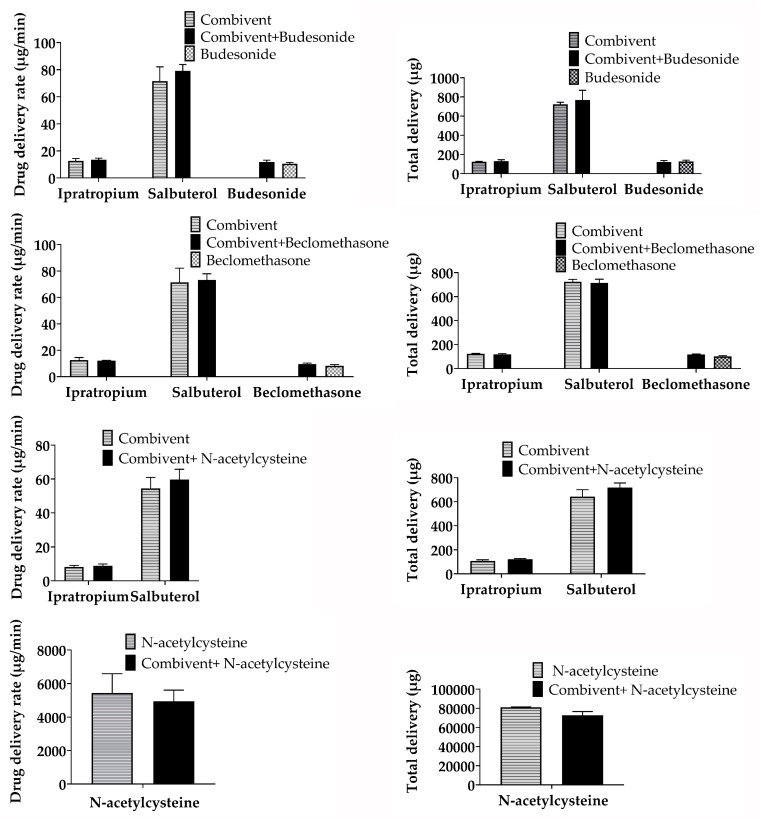
Delivery rate and total delivery of single and mixed inhalation solutions/suspensions.

The delivery rate and total delivery change percentage (%) of the three mixtures compared to single inhalation solutions/suspensions were less than 15%, and the trends of the delivery rate and total delivery were similar (Figure 3). The total time to deliver a single inhalation solution/suspension plus saline was the same for the mixtures. Therefore, the total delivery time was unchanged.

## 4. Discussion

In clinical practice, two or more aerosol inhalation solutions/suspensions are often mixed together for simultaneous inhalation [9]. However, data to address the physico-chemical compatibility and aerosol characteristics of Combivent^®^ and Pulmicort^®^, Clenil^®^, and Fluimucil^®^ do not exist. For the first time, our results indicate that Combivent^®^ is physico-chemically compatible with Pulmicort^®^, Clenil^®^, and Fluimucil^®^. The mixing of Combivent^®^ with Pulmicort^®^, Clenil^®^, and Fluimucil^®^ did not affect the delivery compatibility and particle size distribution of each inhalation solution/suspension.

Combivent^®^ mixed with Pulmicort^®^ and Clenil^®^ appeared stratified after 24 h., However, it could be restored by oscillating the mixture, which is attributed to Pulmicort^®^ and Beclomethason as they are suspensions. After mixing, the pH value changed greatly. This might impact the suspension of budesonide and beclomethasone homogeneity and solubility, etc. These changes might also impact the local dissolution and potential PK/PD. However, we found that the change percentages of pH and the osmotic pressure of the three mixtures at 30 min vs. 0 min and 24 h vs. 0 min were less than 3%. The results of content tests showed that all change percentages were less than 10%. Moreover, a recent study showed that when the pH difference between T0 and T30 was ≤10%, the difference was acceptable [12]. This indicates that Combivent^®^ is physico-chemically compatible with Pulmicort^®^, Clenil^®^, and Fluimucil^®^. In addition, these mixture’s physico-chemical compatibility, particle size, distribution, and delivery compatibility were stable for 24 h at room temperature.

At present, the method of a cascade impactor is the “gold standard” for particle size measurement of inhalants. The change percentage of FPF and MMAD in a mixture was less than 10% based on the collision method. We found that the MMAD of the suspension was more than 6 µm for both single and mixed nebulizers, but it did not change significantly before and after mixing. As a result, it would not affect the aerodynamic compatibility of a single inhalation suspension. The cumulative distribution curves of Budesonide, *N*-acetylcysteine, and Beclomethasone almost coincided, but the trend of Combivent^®^ changed slightly. The X50 measured by laser diffraction method variation was relatively greater for Combivent^®^ mixed with Pulmicort^®^ and Clenil^®^. It may be that the surface tension of the mixed solution changed the particle size [21]. This was also reflected in the delivery compatibility of beclomethasone (the delivery rate and total delivery change percentages were relatively high). The X50 of the mixture with two suspensions tended to increase slightly, which may be due to the instability of suspension droplets. However, the X50 value was still small (around 3 µm) and conformed to the standard of pharmacopeias.

The overall trend of the delivery rate and total delivery before and after nebulizer mixing was the same. The change percentage was less than 15%, which indicates that the mixture delivery compatibility was stable. Compared with other tests, this change percentage was slightly higher, which might have been induced by the respiratory simulation test itself, which has a relatively large error in collection. This is similar to the actual usage in clinical practice. The total time to deliver a single inhalation solution/suspension plus saline is the same for mixtures. Therefore, the total delivery time is unchanged. The nebulizer must be cleaned, dried, and reassembled after use [9]. Therefore, mixing inhalation solutions/suspensions for simultaneous inhalation might simplify the administration procedure.

The results of this study showed that the mixtures’ physico-chemical compatibility, particle size and distribution, and delivery compatibility were stable. Hence, our results suggest that these inhalation solutions/suspensions can be mixed together for simultaneous inhalation. These results are consistent with previously published studies [21,22]. A recent study showed that *N*-acetylcysteine and Ipratropium bromide were stable at 1 h [22]. It is also suggested that the chemical compatibility of Budesonide inhalation suspension mixed with levalbuterol, Salbutamol sulfate, Cromolyn sodium, and Ipratropium bromide was stable within 30 min [23].

The results of our current study have some clinical value and can provide medication suggestions for medical staff and will also help experts revise evidence-based pharmacology in clinical pharmaceutical practice. Although the mixtures were stable for 24 h, there were some Fluctuations in the HPLC baseline after 24 h of mixing. To avoid contamination and bacterial growth of the mixtures, we recommend immediate use. In a follow-up study, factors such as the surface tension, viscosity, and different nebulizer devices need to be investigated further. Finally, clinical studies such as pharmacodynamics and safety should also be considered to ensure the efficacy and safety of simultaneous nebulization compared with sequentially nebulization.

## Figures and Tables

**Table 1:** HPLC gradient conditions of Salbutamol and of Ipratropium bromide and *N*-acetylcysteine.

Time (min)	Flow Rate (mL/min)	A (%)	B (%)
0	1.00	95.0	5.0
10.00	1.00	80.0	20.0
15.00	1.00	80.0	20.0
16.00	1.00	95.0	5.0
25.00	1.00	95.0	5.0

**Table 2:** HPLC gradient conditions of Salbutamol, Ipratropium bromide, and Budesonide and of Salbutamol, Ipratropium bromide, and Beclomethasone.

Time (min)	Flow Rate (mL/min)	A (%)	B (%)
0	1.00	95.0	5.0
10.00	1.00	80.0	20.0
12.00	1.00	80.0	20.0
12.10	1.00	30.0	70.0
20.00	1.00	30.0	70.0
20.10	1.00	95.0	5.0
25.00	1.00	95.0	5.0

**Table 3:** Change percentage of the pH value due to inhalation solution/suspension admixtures at different time points.

Admixture Study	pH of Inhalation Solution/Suspension Prior to Admixing		pH of Admixture			30 min vs. 0 min (%)	24 h vs. 0 min (%)
	Combivent^®^	Other Inhalation Solutions/Suspensions	0 min	30 min	24 h		
Combivent^®^ + Pulmicort^®^	3.55 ± 0.02	4.48 ± 0.06	4.21 ± 0.00	4.25 ± 0.02	4.20 ± 0.03	0.95	0.32
Combivent^®^ + Clenil^®^	3.55 ± 0.02	6.26 ± 0.03	3.73 ± 0.04	3.73 ± 0.06	3.80 ± 0.03	0.18	1.97
Combivent^®^ + Fluimucil^®^	3.55 ± 0.02	6.62 ± 0.01	6.52 ± 0.01	6.49 ± 0.02	6.37 ± 0.03	0.41	2.25

**Table 4:** Change percentage of the osmotic pressure due to inhalation solution/suspension admixtures at different time points.

Admixture Study	Osmotic Pressure of Inhalation Solution/Suspension Prior to Admixing (mOsm/kg)		Osmotic Pressure of Admixture (mOsm/kg)			30 min vs. 0 min (%)	24 h vs. 0 min (%)
	Combivent^®^	Other Inhalation Solutions/Suspensions	0 min	30 min	24 h		
Combivent^®^ + Pulmicort^®^	276.67 ± 0.47	270.33 ± 0.47	282.67 ± 0.94	284.00 ± 2.16	278.00 ± 0.00	0.47	1.65
Combivent^®^ + Clenil^®^	276.67 ± 0.47	283.00 ± 0.82	290.00 ± 1.63	287.33 ± 0.94	289.67 ± 0.94	0.92	0.11
Combivent^®^ + Fluimucil^®^	276.67 ± 0.47	1312.67 ± 2.05	813.67 ± 2.87	818.33 ± 0.47	807.33 ± 1.89	0.57	0.78

**Table 5:** Change percentage of the content due to inhalation solution/suspension admixtures at different time points.

Admixture Study	Active Ingredients	0 min Single vs. Admixture (%)	30 min Single vs. Admixture (%)	24 h Single vs. Admixture (%)	30 min vs. 0 min (%)	24 h vs. 0 min (%)
Combivent^®^ + Pulmicort^®^	Ipratropium	1.7	0.4	3.6	1.0	1.7
	Salbutamol	5.9	4.1	7.6	1.2	0.9
	Budesonide	5.9	9.4	9.5	2.4	4.7
Combivent^®^ + Clenil^®^	Ipratropium	1.8	2.9	6.3	1.5	0.8
	Salbutamol	2.6	3.7	6.5	1.7	1.3
	Beclomethasone	3.3	2.8	7.1	3.0	1.3
Combivent^®^ + Fluimucil^®^	Ipratropium	6.7	6.9	2.0	0.0	8.7
	Salbutamol	1.0	1.4	1.7	0.0	2.2
	*N*-Acetylcysteine	6.9	6.0	3.3	0.0	0.4

**Table 6:** MMAD and FPF and their change percentages for the single and mixed inhalation solutions/suspensions.

Admixture Study	Active Ingredients	MMAD (µm)	FPF (%)	MMAD Change Percentage (%)	FPF Change Percentage (%)
Combivent^®^	Ipratropium	5.01 ± 0.05	48.64 ± 0.45		
	Salbutamol	4.98 ± 0.07	48.86 ± 0.56		
Pulmicort^®^	Budesonide	6.54 ± 0.22	32.10 ± 2.32		
Clenil^®^	Beclomethasone	6.96 ± 0.02	27.16 ± 0.26		
Fluimucil^®^	*N*-Acetylcysteine	4.94 ± 0.07	49.98 ± 0.71		
Combivent^®^ + Pulmicort^®^	Ipratropium	4.90 ± 0.08	49.59 ± 0.78	2.21	1.92
	Salbutamol	5.40 ± 0.03	45.33 ± 0.27	2.63	2.42
	Budesonide	6.25 ± 0.17	34.99 ± 1.56	4.57	8.26
Combivent^®^ + Clenil^®^	Ipratropium	5.45 ± 0.03	45.00 ± 0.37	8.07	8.09
	Salbutamol	5.40 ± 0.03	45.33 ± 0.27	7.88	7.80
	Beclomethasone	6.99 ± 0.20	27.48 ± 1.55	0.39	1.19
Combivent^®^ + Fluimucil^®^	Ipratropium	4.70 ± 0.05	51.87 ± 0.64	6.50	6.23
	Salbutamol	4.85 ± 0.09	50.07 ± 0.87	7.03	6.71
	*N*-Acetylcysteine	4.78 ± 0.08	51.71 ± 0.86	3.35	3.34

**Table 7:** X10, X50, X90 and span of single and mixed inhalation solutions/suspensions.

Admixture Study	X10 (µm)	X50 (µm)	X90 (µm)	Span
Combivent^®^	0.72 ± 0.04	2.54 ± 0.22	8.38 ± 0.41	3.03 ± 0.11
Pulmicort^®^	0.69 ± 0.02	2.66 ± 0.08	8.96 ± 0.01	3.12 ± 0.10
Clenil^®^	0.78 ± 0.01	2.88 ± 0.04	8.96 ± 0.07	2.84 ± 0.02
Fluimucil^®^	0.65 ± 0.04	2.51 ± 0.03	8.26 ± 0.16	3.03 ± 0.03
Combivent^®^ + Pulmicort^®^	0.82 ± 0.11	3.20 ± 0.01	9.85 ± 0.24	2.82 ± 0.04
Combivent^®^ + Clenil^®^	0.76 ± 0.01	3.27 ± 0.09	9.87 ± 0.16	2.79 ± 0.05
Combivent^®^ + Fluimucil^®^	0.86 ± 0.01	2.91 ± 0.03	8.77 ± 0.04	2.72 ± 0.02
